# Pneumothorax complicating isolated clavicle fracture

**DOI:** 10.11604/pamj.2015.21.202.6796

**Published:** 2015-07-20

**Authors:** Redouane Hani, Badr Ennaciri, Idriss Jeddi, Ahmed El Bardouni, Mustapha Mahfoud, Mohamed Saleh Berrada

**Affiliations:** 1 Department of Orthopedic Surgery and Traumatology, Mohamed V Souissi University, Rabat, Morocco

**Keywords:** Clavicle, fracture, pneumothorax, chest drain

## Abstract

Isolated clavicle fractures are among the commonest of traumatic fractures in the emergency department. Complications of isolated clavicle fractures are rare. Pneumothorax has been described as a complication of a fractured clavicle only rarely in English literature. In all the reported cases, the pneumothorax was treated by a thoracostomy and the clavicle fracture was treated conservatively. In our case, the pneumothorax required a chest drain insertion and the clavicle fracture was treated surgically with good result.

## Introduction

Fractures of the clavicle are common injuries in the emergency department, representing approximately 4% of all fractures. These fractures are comparatively easy to manage and typically heal with routine immobilization. Pneumothorax as a result of a clavicle fracture is very rare, but potentially lethal complication. It has only been reported few times in English literature. In all earlier reported cases, the pneumothorax was treated by thoracostomy and the clavicle fracture was treated conservatively. We describe a rare case of closed fracture of the left clavicle complicated with a significant pneumothorax. The clavicle fracture was treated surgically and the pneumothorax conservatively with good result.

## Patient and observation

**Figure 1:**
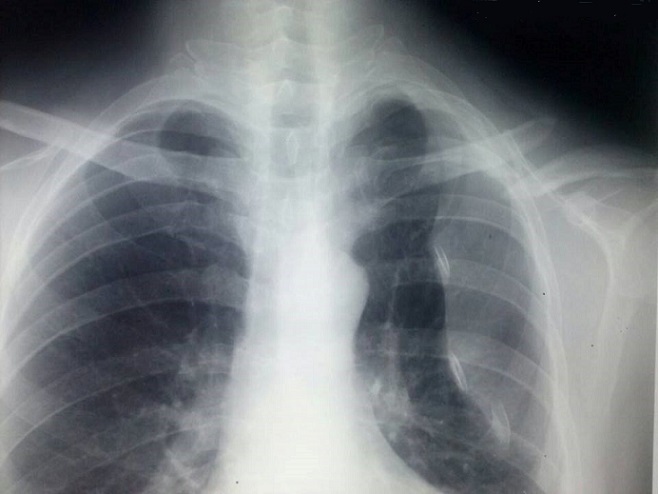
Chest radiograph taken two hours after the injury, showing left fractured clavicle and pneumothorax

**Figure 2:**
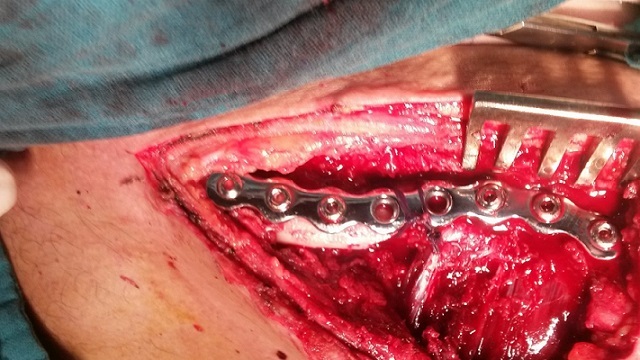
Open reduction and plate osteosynthesis of the clavicle fracture

**Figure 3:**
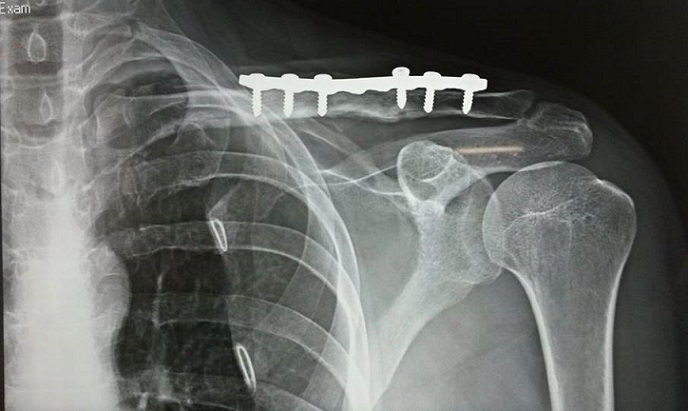
Chest radiographs showing callus formation of the fractured clavicle 14 months after the injury with no recurrence of pneumothorax

A 28-year-old male professional cyclist presented to the emergency department having fallen off his bicycle. On presentation he complained of a painful left shoulder and on inspiration. There was no relevant medical history. The patient presented without clinical distress. Breath sounds and percussion notes were normal on both sides with no visible injuries of the chest wall. The patient was hemodynamically stable and clinically, there was an evident fracture of the left clavicle with intact skin and no neurovascular abnormalities. Radiographs of the clavicle showed a displaced midshaft fracture of the left clavicle with a left-sided pneumothorax (Figure 1). The patient continued to complain of pain on inspiration. A chest radiograph revealed a 50% pneumothorax on the same side as the clavicular fracture. There were no rib fractures. The pneumothorax was treated by the insertion of a chest drain under local anesthesia. The lung had expanded completely two days after the injury and the drain was removed. The fractured clavicle was treated surgically. Internal fixation with plate osteosynthesis was performed (Figure 2). Regular follow-up chest radiographs showed no recurrence of the pneumothorax, and at 6 weeks post-op the patient had full range of motion and presented a well healing fracture with callus formation evident by radiographic images (Figure 3).

## Discussion

The clavicle is one of the most commonly fractured bones, accounting for up to 4% of all fractures[1]. These fractures are comparatively easy to manage and typically heal with routine immobilization. Anatomically, the apex of the lung lies behind and above the medial one third of the clavicle, with the anterior scalene muscle, brachial plexus, and subclavian vessel interferences. However, there are few actual reported cases of isolated clavicle fractures causing a pneumothorax, with subclavian vessel injury and brachial plexus paresis being mentioned in the literature as other rare complications[1–3]. The overall incidence of these complications is below 1-3%, which includes cases with first rib and scapular fractures in addition to the clavicular fracture [4]. Most clavicular fractures result from a fall on an ipsilateral shoulder. Other mechanisms of injury include direct blows and falls on an ipsilateral outstretched hand. Yates suggested that complications, including pneumothorax, following this injury were more common when due to direct trauma of the shoulder [1]. Nevertheless operative treatment is indicated in cases of clavicle fractures with an initial shortening of more than 15 mm, a risk of perforation of the skin, neurovascular complications and pseudarthrosis. Due to the current trend of nonunion rates in nonsurgical management, open reduction and internal fixation (ORIF) has become readily accepted in clavicular fracture management [5]. In our case, the patient complained of pain on inspiration, and radiographs of the clavicle showed a displaced midshaft fracture of the left clavicle with a significant pneumothorax, requiring emergency treatment by chest drain insertion under local anesthesia. The fractured clavicle was treated by internal fixation with plate osteosynthesis without complications. Regular follow-up chest radiographs showed no recurrence of the pneumothorax, and at six weeks, the clavicle fracture had clinically united.

## Conclusion

Although clavicle fractures are common injuries, rarely requiring more than conservative treatment on an out-patient basis, pneumothorax should be considered as a potential complication and must be excluded by appropriate clinical examination and radiographs.
